# Relationship Between Alcohol Consumption And Clinical Profile in The Tunisian Population

**DOI:** 10.1192/j.eurpsy.2026.10855

**Published:** 2026-07-31

**Authors:** I. Mannoubi, M. Turki, S. Kotti, M. A. Megdiche, N. Halouani, S. Ellouze, J. Aloulou

**Affiliations:** Psychiatry B, Hedi Chaker hospital, Sfax, Tunisia

## Abstract

**Introduction:**

Alcohol consumption remains a serious public health challenge, affecting various clinical dimensions. Understanding its impact on individuals’ clinical profiles is essential to optimize prevention and management strategies.

**Objectives:**

This study aimed to analyze the association between alcohol consumption clinical characteristics.

**Methods:**

A descriptive and analytical cross-sectional study was carried out among 161 participants through an anonymous online survey administered via Google Form. Collected data included weight, height, body mass index (BMI), and personal medical and psychiatric history. We used the Alcohol Use Disorders Identification Test (AUDIT) to assess alcohol use.

**Results:**

Participants had a mean a mean BMI of 24.17 kg/m^2^ (16.04–39.04), with most individuals within the normal weight range. Psychiatric disorders were reported in 19.3% (N = 31) of participants, with anxiety and depressive disorders being the most common (6.8% each), followed by bipolar disorder (5.6%) and eating disorders (1.2%). Regarding medical history, 23% (N=37) of participants had personal medical conditions, predominantly hepatic (8.1%), endocrine (4.3%), and respiratory (3.7%) diseases.Analysis of AUDIT scores showed that obese participants had the highest mean scores (5.36), with a trend toward significance (p = 0.0689). AUDIT scores were also higher in participants with psychiatric histories, with addictive disorders being significantly associated with higher scores (p = 0.03). Alcohol dependence was significantly more frequent among participants with anxiety disorders (p=0.001), bipolar disorder (p=0.004), and eating disorders (p=0.003). Participants with somatic diseases had slightly higher mean AUDIT scores (3.35 vs. 2.19) and a trend toward a higher proportion of dependence (8.1% vs. 0.8%, p = 0.0571).

**Conclusions:**

This study highlights the close relationship between alcohol use, psychiatric disorders, and certain medical conditions. These findings underscore the importance of systematic screening for alcohol use in individuals with psychiatric or medical conditions, in order to guide prevention and tailored management strategies.

**Disclosure of Interest:**

None Declared

